# Long‐Acting PrEP for People With High Vulnerability to HIV Acquisition in Brazil: A Cost‐Effectiveness Analysis

**DOI:** 10.1002/jia2.70116

**Published:** 2026-05-14

**Authors:** Wanyi Chen, Paula M. Luz, Anjali Srinivasan, Beatriz Grinsztejn, Justine A. Scott, Anne M. Neilan, Valdiléa G. Veloso, Caitlin M. Dugdale, Kenneth A. Freedberg

**Affiliations:** ^1^ Medical Practice Evaluation Center, Massachusetts General Hospital Boston Massachusetts USA; ^2^ Instituto Nacional de Infectologia Evandro Chagas, Fundação Oswaldo Cruz de Janeiro Brazil; ^3^ Harvard Medical School Boston Massachusetts USA; ^4^ Division of Infectious Diseases, Massachusetts General Hospital Boston Massachusetts USA; ^5^ Center for AIDS Research, Harvard University Cambridge Massachusetts USA; ^6^ Division of General Academic Pediatrics, Massachusetts General Hospital Boston Massachusetts USA; ^7^ Division of General Internal Medicine Massachusetts General Hospital Boston Massachusetts USA; ^8^ Department of Health Policy and Management Harvard T.H. Chan School of Public Health Boston Massachusetts USA

**Keywords:** Brazil, cost‐effectiveness analysis, HIV, long‐acting PrEP, MSM, TGW

## Abstract

**Introduction:**

In Brazil, men who have sex with men (MSM) and transgender women (TGW) remain heavily affected by HIV. Long‐acting pre‐exposure prophylaxis (LA PrEP) with injectable cabotegravir (CAB‐LA) or lenacapavir (LEN‐LA) is more effective at preventing HIV acquisition than oral PrEP. Our objective was to assess the potential clinical and economic impact of offering CAB‐LA or LEN‐LA to MSM and TGW with high vulnerability to HIV acquisition in Brazil and determine the maximum cost at which they would be cost‐effective.

**Methods:**

We used the CEPAC microsimulation model of HIV prevention and treatment to evaluate two strategies for MSM and TGW aged 18−49: (1) *SOC*: standard‐of‐care oral PrEP at current coverage, and (2) *SOC+LA*: offering oral and LA PrEP (either CAB‐LA or LEN‐LA). Input parameters are derived from Brazil‐based data from 2010 to 2024 and published studies: HIV incidence (%/year, MSM: 3.4 [age 18−29 years], 1.1 [30−49 years]; TGW: 5.0 [18−29 years], 1.7 [30−49 years]), relative risk reduction, LA versus oral PrEP (66% [CAB‐LA]; 89% [LEN‐LA]), PrEP coverage (20% [oral PrEP]; 20% [LA PrEP]) and oral PrEP cost (programmatic+drug = $207/year). Outcomes include lifetime HIV risk, life expectancy (LE) and incremental cost‐effectiveness ratio (ICER) of *SOC+LA* versus *SOC* in 2024 USD/year of life saved (YLS). We identified the maximum LA PrEP cost with ICER below the established Brazilian willingness‐to‐pay threshold of $8740/YLS.

**Results:**

Compared to *SOC, SOC+CAB‐LA* would decrease MSM lifetime HIV risk from 21.4% to 16.8%, increase undiscounted LE from 39.0 to 39.4 years. For TGW, *SOC+CAB‐*LA would decrease lifetime HIV risk from 29.5% to 23.4%, increase LE from 36.0 to 36.9 years. Results for *SOC+LEN‐LA* would be similar to *SOC+CAB‐LA*. SOC+LA would remain cost‐effective for MSM at cost below $710/year for CAB‐LA and $740/year for LEN‐LA. Findings are most sensitive to LA PrEP cost, HIV incidence, and whether and by how much LA PrEP increases coverage.

**Conclusions:**

Offering LA PrEP with cabotegravir or lenacapavir in addition to oral PrEP for MSM and TGW in Brazil could markedly improve clinical outcomes and be cost‐effective at ∼$700/year. Cost agreements are critical to ensure these prevention options are accessible in high‐incidence settings.

## 1 Introduction

In Brazil, the HIV epidemic continues unabated among men who have sex with men (MSM) and transgender women (TGW). In 2023, over 500,000 MSM and 400,000 TGW were living with HIV across the nation [1, 2, 3, 4]. Recent annualized HIV incidence estimates among MSM/TGW range between 2.6% and 7.4% [2, 5, 6, 7, 8].

Brazil's public health system introduced oral tenofovir/emtricitabine (TDF/FTC) pre‐exposure prophylaxis (PrEP) in 2017 to bolster HIV prevention [9, 10]. However, coverage remains well below the 50% UNAIDS goal for MSM/TGW [11, 12, 13, 14]. Barriers to oral PrEP use include stigma, discrimination, side effect concerns and desire not to take a daily pill [14, 15]. These barriers contribute to low PrEP initiation, poor adherence (i.e. taking PrEP as scheduled to maintain adequate drug levels) and suboptimal PrEP persistence (i.e. longitudinal engagement in PrEP programmes).

Two new long‐acting PrEP (LA PrEP) products may help overcome these barriers and offer a promising, highly effective alternative to oral PrEP. The HPTN 083 and PURPOSE‐2 trials found that LA PrEP in the form of long‐acting cabotegravir (CAB‐LA) and long‐acting lenacapavir (LEN‐LA) were superior to oral TDF/FTC in preventing HIV acquisition among MSM/TGW [16, 17]. Studies have shown that LA PrEP was preferred over oral PrEP among MSM/TGW in Brazil [15, 18, 19, 20]. This suggests that LA PrEP can potentially overcome adherence and persistence difficulties inherent to oral regimens [21].

While CAB‐LA and LEN‐LA were approved for use as PrEP in the United States in 2021 and 2025 [22, 23], neither is yet widely available in low‐ and lower‐middle‐income countries (LMICs), due largely to drug costs [24]. Although the costs of LA PrEP products remain undetermined, ViiV has proposed a cost of $1440/year for CAB‐LA [25]. International agreements would permit generic production of CAB‐LA in 90 LMICs, offering promise for affordability [26]. However, due to its upper middle‐income country status, Brazil is not eligible for this negotiated cost [25, 27]. Our objective was to assess the potential long‐term clinical and economic impact of offering CAB‐LA or LEN‐LA to MSM and TGW with high vulnerability to HIV acquisition in Brazil and determine the maximum cost threshold at which providing LA PrEP would be cost‐effective compared with providing only oral PrEP.

## 2 Methods

### 2.1 Analytic Overview

Using the Cost‐Effectiveness of Preventing AIDS Complications (CEPAC) model, we projected the clinical, epidemiologic and economic impact of LA PrEP for MSM and TGW aged 18−49 in Brazil, using Brazil‐specific input data from 2010 to 2024 [28, 29, 30]. We compared two strategies: (1) *SOC*: standard‐of‐care oral PrEP (TDF/FTC) at current coverage, and (2) *SOC+LA*: continuing standard‐of‐care oral PrEP with additional CAB‐LA or LEN‐LA. We modelled MSM and TGW separately; for each group, we compared *SOC+CAB‐LA* or *SOC+LEN‐LA* to *SOC*. Outcomes included 10‐year and lifetime HIV risk, life expectancy (LE), lifetime costs and incremental cost‐effectiveness ratios (ICERs) in 2024 USD per year of life saved (YLS). We discounted LE and costs at 5%/year [31]. We used a healthcare sector payer perspective (Brazil's national health system), including PrEP drug and programme costs and downstream HIV care costs for those who acquire HIV. We identified the maximum LA PrEP costs for *SOC+LA* to be cost‐effective, with an ICER below a willingness‐to‐pay threshold of $8740/YLS (87% of per capita 2024 GDP of $10,040), as per Brazil's National Committee for the Incorporation of Technologies (CONITEC) or below 50% of per capita GDP [32, 33]. Because MSM constitute a larger target population than TGW, we anchored cost thresholds to the MSM cohort to reflect population‐level impact and payer‐level feasibility. We report ICERs and maximum costs incorporating the benefit of reduced primary transmissions, defined as one generation of new HIV infections transmitted from members of the initial cohorts to others outside the initial cohorts. We additionally report more pessimistic estimates that exclude indirect transmission benefits for the base case analysis.

### 2.2 CEPAC Model Structure and Inputs

CEPAC is a validated and widely published microsimulation model of HIV testing, prevention and treatment [34, 35, 36]. Model code supporting this analysis (version 50dMDLT) is publicly available at https://github.com/MGH‐MPEC/CEPAC. Input data are described below and in Table 1.

**TABLE 1:** Select base case inputs for a cost‐effectiveness analysis of LA PrEP for MSM and TGW in Brazil.

	Value	Range	Reference
**Baseline cohort characteristics**			
Initial age distribution, %			[5]
18−24 years	33		
25−29 years	34		
30−49 years	33		
MSM annual HIV incidence by age, %			
18−29 years	3.37	1.69−6.74	[5]
30−49 years	1.11	0.56−2.22	[5]
TGW annual HIV incidence by age, %			
18−29 years	5.06	2.53−10.1	[5, 7]
30−49 years	1.67	0.83−3.33	[5, 7]
CD4 count at ART initiation, cells/µL, mean (SD)			Derived from [36], INI^a^
MSM	391 (271)		
TGW	433 (313)		
**PrEP characteristics**			
PrEP effectiveness, %			
Oral PrEP	75	38−86	[42, 43]
CAB‐LA	92	85−96	Derived from [16, 42, 43, 75]
LEN‐LA	97	87−100	Derived from [42, 43, 75, 76]
PrEP coverage, %			
Oral PrEP	20	10−30	[13, 14, 44]
CAB‐LA	20	10−30	Assumption
LEN‐LA	20	10−30	Assumption
**PrEP programme costs**			
Drug cost, $/year			
Oral PrEP	204.56		[60]
CAB‐LA	600	200−1200	Assumption
LEN‐LA	600	200−1200	Assumption
PrEP provider time costs			
Initiation visit cost, $			[60, 61, 62, 63]
Oral PrEP	5.49		
CAB‐LA	7.38		
LEN‐LA	7.38		
Follow‐up visit cost, $/visit			[60, 61, 62, 63]
Oral PrEP	3.03		
CAB‐LA	4.92		Assumption
LEN‐LA	4.92		Assumption
HIV test cost, $/test	0.18		[60, 63]
**HIV‐related care costs**			
Routine care cost, $/year (range by CD4)	38.08–428.99		[28, 63, 65, 66, 77]
ART cost, $/year			
First‐line (TLD)	434.60		[60, 63]
Second‐line (DRV/r+2NRTIs)	904.07		[60, 63]

Abbreviations: ART, antiretroviral therapy; CAB‐LA, cabotegravir long‐acting; DRV/r, darunavir/ritonavir; HIV, human immunodeficiency virus; INI, Instituto Nacional de Infectologia Evandro Chagas; LA PrEP, long‐acting pre‐exposure prophylaxis; LEN‐LA, lenacapavir long‐acting; LTFU, loss‐to‐follow‐up; MSM, men who have sex with men; NRTIs, nucleoside reverse transcriptase inhibitors; PrEP, pre‐exposure prophylaxis; PY, person‐years; SD, standard deviation; TGW, transgender women; TLD, tenofovir disoproxil, lamivudine and dolutegravir.

^a^ Derived from the HIV Clinical Cohort at INI/FIOCRUZ among those enrolled between 2010 and 2019 with follow‐up data until the end of 2020.

#### 2.2.1 Cohort Characteristics

Simulated cohorts’ starting age distribution reflects the ImPrEP seroincidence study in Brazil: 18−24 years (33%), 25−30 years (34%) and 30−49 years (33%) [5]. Incidence without PrEP is from Brazil‐based sources [5, 7]: 3.37%/year (MSM 18−29), 1.11%/year (MSM 30−49), 5.06%/year (TGW 18−29) and 1.67%/year (TGW 30−49). HIV incidence above age 50 was not modelled. We calibrated mean CD4 count at HIV acquisition (615 cells/µL MSM, 690 cells/µL TGW), given a decline of 6 cells/µL/year to match observed age (31.5 years) and CD4 counts (391 cells/µL MSM, 433 cells/µL TGW) at antiretroviral therapy (ART) initiation in Brazil [28, 36, 37, 38].

#### 2.2.2 HIV Primary Transmissions

HIV transmission probability is a function of HIV RNA level, which depends on infection stage and ART response [39, 40, 41]. Supporting Information Methods provide details for transmission rates calibration and incorporating indirect transmission benefits into ICER calculations. Transmission rates are 0.0−100.7 per 100 person‐years for MSM and 0.0−8.0 per 100 person‐years for TGW, by disease stage and HIV RNA (Supporting Information Table A1) [39, 40, 41].

#### 2.2.3 PrEP Characteristics

##### 2.2.3.1 PrEP Effectiveness

We modelled oral PrEP effectiveness as the product of efficacy (96% reduction in HIV risk with ≥4 doses/week) [42] and adherence (78% taking ≥4 pills/week) [43], resulting in 75% effectiveness. We applied observed relative risk reductions with LA versus oral PrEP from the HPTN 083 and PURPOSE trials to model LA PrEP effectiveness, reflecting combined effects of adherence, persistence and biological efficacy [16, 17]. This resulted in effectiveness of 92% (95% CI 85%–96%) for CAB‐LA and 97% (95% CI 87%–100%) for LEN‐LA (Supporting Information Methods).

##### 2.2.3.2 PrEP Coverage

We define PrEP coverage as the proportion of MSM/TGW without HIV enrolled in a PrEP programme at a given time. Though individuals in a PrEP programme can discontinue and restart, we assume constant PrEP coverage to reflect the steady‐state population‐level fraction of the population that would be using PrEP. Individuals become ineligible for PrEP when reaching a stopping age or upon HIV diagnosis.

Informed by cross‐sectional data from Brazil, oral PrEP coverage was 20% among MSM and TGW aged 18−49, in *SOC* and *SOC+LA* strategies [13, 14, 44]. In the base case with additive coverage, we assume the same coverage of 20% for LA PrEP (CAB‐LA or LEN‐LA) in addition to 20% oral PrEP coverage. We assessed more pessimistic coverage scenarios in sensitivity and scenario analyses.

#### 2.2.4 HIV Testing and Linkage to Care

Individuals are tested for HIV via “background” testing at 2%/month based on Brazil data [45], and upon presentation to care with an opportunistic infections (OI). Individuals on PrEP experience more frequent testing [10, 46]. With *SOC*, HIV testing is every 3 months per oral PrEP guidelines [47]. With CAB‐LA, HIV testing occurs at baseline, month 1 and thereafter every 2 months per WHO guidelines [16, 48]. With LEN‐LA, HIV testing occurs at baseline and then every 6 months [49].

#### 2.2.5 HIV Treatment

Once diagnosed and linked to care, people living with HIV infection (PWH) receive ART per Brazil guidelines, starting with a dolutegravir (DTG)‐based regimen, followed by a protease inhibitor (PI)‐based regimen upon virologic failure on first‐line ART [50]. Regimen‐specific rates of initial virologic suppression and subsequent rates of virologic failure are derived from the literature (Table 1) [51, 52, 53, 54].

#### 2.2.6 Development of Resistance While Using LA PrEP

From observational data [55], we assumed that 31% of individuals who acquired HIV while using CAB‐LA would develop integrase strand transfer inhibitor (INSTI) resistance, resulting in failure to suppress on DTG‐based regimens and subsequent use of PI‐based ART [55, 56, 57]. We assume that individuals who acquire HIV while using LEN‐LA may develop capsid inhibitor‐resistance and would then use DTG‐based regimens [58].

#### 2.2.7 Costs

Oral PrEP programme costs include TDF/FTC ($205/year), HIV testing ($0.20/test, 4x/year) and provider time for test administration ($3.00/test). Because neither CAB‐LA nor LEN‐LA is yet available in Brazil, we assume a base‐case drug cost of $600/year, about three times oral PrEP cost and consistent with cost‐effective LA‐PrEP costs from South African studies [24, 59]. We vary costs from $200/year to $1200/year to assess the impact on conclusions. We assume provider time for LA PrEP injection administration is 10 minutes more than with oral PrEP, resulting in programmatic costs of $20 at initiation, including HIV testing, and $4.90 at each follow‐up injection [60, 61, 62, 63]. Costs for ART, OI treatment, CD4 count, HIV RNA monitoring and routine HIV‐related care are from the Instituto Nacional de Infectologia Evandro Chagas (INI) cohort and Brazilian Ministry of Health (Table 1) [60, 63, 64, 65, 66].

### 2.3 Sensitivity Analysis

We examined the impact of varying key inputs on cost‐effectiveness results. Inputs varied include: oral PrEP adherence, LA PrEP effectiveness, LA PrEP costs, HIV incidence among MSM and TGW, PrEP stopping age, discount rate, prevalence of INSTI‐based resistance when failing CAB‐LA, and oral and LA PrEP coverage reflecting additive and substitutive scenarios, with a maximum oral versus LA PrEP ratio of 1:4 based on a Brazil CAB implementation study [67]. In multiway sensitivity analysis, we simultaneously varied inputs most influential in one‐way sensitivity analysis.

### 2.4 Scenario Analysis

Under a more pessimistic scenario with partial substitution coverage, we assumed 5% of individuals switch from oral PrEP to LA PrEP in the *SOC+LA* strategy, resulting in 15% oral PrEP and 15% LA PrEP coverage, or 30% total coverage.

## 3 Results

### 3.1 Base Case

#### 3.1.1 Clinical

Infection risk for MSM would decrease from 21.4% to 16.8% with *SOC+CAB‐LA*, and to 16.5% with *SOC+LEN‐LA* over a lifetime horizon (Table 2). Undiscounted LE for MSM would increase from 39.0 to 39.4 years with *SOC+CAB‐LA*, and further to 39.5 years with *SOC+LEN‐LA*. The clinical benefit of *SOC+LA* over *SOC* would be greater for TGW compared to MSM, with either CAB‐LA or LEN‐LA.

**TABLE 2:** Base case results of a cost‐effectiveness analysis of LA PrEP for MSM and TGW in Brazil with 40% total PrEP coverage (additive coverage).

Strategy	Undisc. LE, year	Disc. LE, year	10‐year infection risk, %	Lifetime infection risk^a^, ^c^, %	Undisc. costs, $	Disc. costs, $	ICER^b^, $/YLS
**MSM**							
LA PrEP option = cabotegravir							
*SOC*	39.0	16.2	14.0	21.4	2800	1060	—
*SOC+CAB‐LA*	39.4	16.3	11.0	16.8	4780	2400	7300
LA PrEP option = lenacapavir							
*SOC*	39.0	16.2	14.0	21.4	2800	1060	—
*SOC+LEN‐LA*	39.5	16.3	10.8	16.5	4670	2350	6800
**TGW**							
LA PrEP option = cabotegravir							
*SOC*	36.0	15.7	20.0	29.5	2660	1080	—
*SOC+CAB‐LA*	36.9	15.8	15.8	23.4	4620	2390	6790
LA PrEP option = lenacapavir							
*SOC*	36.0	15.7	20.0	29.5	2660	1080	—
*SOC+LEN‐LA*	36.9	15.8	15.5	23.0	4530	2340	6360

Abbreviations: Disc., discounted; ICER, incremental cost‐effectiveness ratio; LA PrEP, long‐acting pre‐exposure prophylaxis; LE, life expectancy; MSM, men who have sex with men; SOC, standard‐of‐care using oral PrEP at 20% coverage; SOC+LA, strategy providing both oral PrEP (coverage 20%) and LA PrEP (coverage 20%) using CAB‐LA or LEN‐LA; TGW, transgender women; Undisc., undiscounted; YLS, years of life saved.

^a^ We did not model HIV incidence above age 50.

^b^ This is the discounted ICER of SOC+LA compared to SOC, where the cost of CAB‐LA or LEN‐LA is $600/year, adjusted to include the benefit of reduced primary transmissions. Without adjusting for transmissions, the discounted ICERs would be $13,080/YLS (MSM) and $7280/YLS (TGW) for SOC+CAB‐LA versus SOC, or $12,540/YLS (MSM) and $6840/YLS (TGW) for SOC+LEN‐LA versus SOC.

^c^ The number needed to treat (NNT) to prevent one case of HIV over 10 years is calculated for each group as the number of HIV cases expected over 10 years with no PrEP compared to SOC (oral PrEP), CAB‐LA or LEN‐LA. For MSM, the NNT for SOC is 8, for CAB‐LA 7 and for LEN‐LA 6 (Supporting Information Methods). For TGW, the numbers are lower due to the higher incidence of HIV: for SOC, the NNT is 6, for CAB‐LA 5 and for LEN‐LA 4. If we included the second “generation” of cases prevented, the NNT would be slightly lower for all PrEP strategies.

#### 3.1.2 Costs, Cost‐Effectiveness and Maximum Cost Incorporating Transmission Benefits

For MSM, per‐person undiscounted lifetime costs would be $2800 with *SOC*. At an LA PrEP cost of $600/year, per‐person undiscounted lifetime costs would increase to $4780 with *SOC+CAB‐LA* and $4620 with *SOC+LEN‐LA*. Incorporating transmission benefits at this cost, *SOC+LA* would be cost‐effective compared to *SOC* (discounted ICER: $7300/YLS, *SOC+CAB‐LA*; $6800/YLS, *SOC+LEN‐LA*), given the Brazil‐defined willingness‐to‐pay threshold of $8740/YLS. For TGW, *SOC+LA* would be cost‐effective with ICERs of *SOC+LA* versus *SOC* of $6790/YLS with CAB‐LA and $6360/YLS with LEN‐LA.

*SOC+LA* would remain cost‐effective compared to *SOC* for MSM at a cost below $710/year if using CAB‐LA and $740/year with LEN‐LA (Supporting Information Figure A1). *SOC+LA* would be cost‐effective for MSM at a willingness‐to‐pay threshold of $5020/YLS (50% GDP per capita) if it cost less than $430/year with CAB‐LA and $470/year with LEN‐LA.

#### 3.1.3 Costs, Cost‐Effectiveness and Maximum Cost Excluding Transmission Benefits

At an LA PrEP cost of $600/year, when benefits of primary transmissions were not accounted for, *SOC+LA* would not be cost‐effective compared to *SOC* for MSM (ICER: $13,080/YLS, *SOC+CAB‐LA*; $12,540/YLS, *SOC+LEN‐LA*), given the Brazil‐defined willingness‐to‐pay threshold of $8740/YLS. For TGW, *SOC+LA* would be cost‐effective (ICER: $7280/YLS, *SOC+CAB‐LA*; $6840/YLS, *SOC+LEN‐LA)*. *SOC+LA* would be cost‐effective compared to *SOC* for MSM at a cost below $410/year if using CAB‐LA and $430/year with LEN‐LA.

### 3.2 One‐Way Sensitivity Analysis Incorporating Transmission Benefits

**FIGURE 1:**
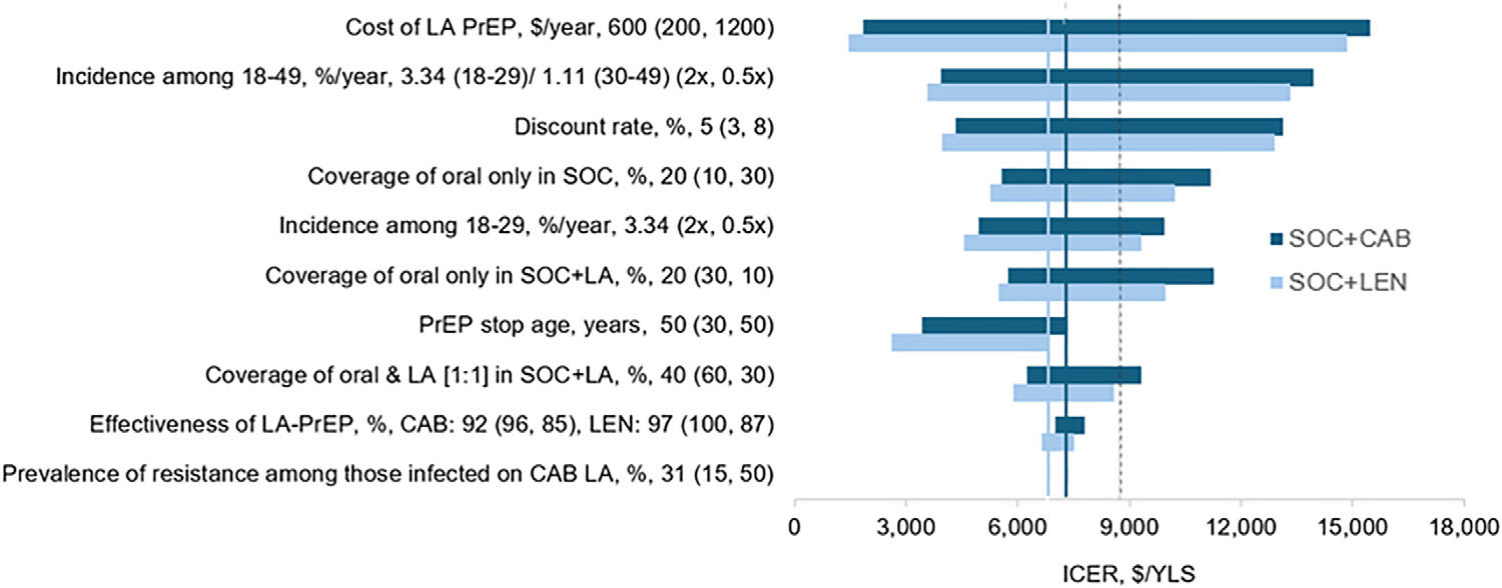
One‐way sensitivity analyses of LA PrEP cost‐effectiveness for MSM in Brazil. The tornado diagram displays the impact of varying individual parameters on the change in ICERs (*SOC+CAB‐LA* [dark blue] and *SOC+LEN‐LA* [light blue] vs. *SOC*). Each horizontal bar displays the range of ICERs that result from varying a single parameter. For each parameter, the base case value is listed first, followed by the range in the parentheses. The base case values would lead to the thin vertical lines through the diagram, where the ICERs are $7300/YLS (*SOC+CAB‐LA*) and $6800/YLS (*SOC+LEN‐LA*). The left value in the parentheses would lead to the lower ICER from the sensitivity analysis, and the right value would lead to the higher ICER. A longer bar reflects a greater change in ICER as the parameter was varied. The black dotted vertical line at $8740/YLS marks the willingness‐to‐pay threshold. The bars that extend to the right of this line mark where *SOC+LA* would not be cost‐effective compared to *SOC*, while the bars that extend to the left mark where *SOC+LA* would be cost‐effective compared to *SOC*. Abbreviations: CAB‐LA, cabotegravir long‐acting; ICER, incremental cost‐effectiveness ratio; LA PrEP, long‐acting pre‐exposure prophylaxis; LEN‐LA, lenacapavir long‐acting; MSM, men who have sex with men; YLS, years of life saved.

Among MSM, CAB‐LA cost was most influential on ICERs. As cost ranged from $200/year to $1200/year, the ICER of *SOC+CAB‐LA* versus *SOC* would increase from $1850/YLS to $15,470/YLS, exceeding the willingness‐to‐pay threshold of $8740/YLS at higher costs (Figure 1). The next most influential parameters were HIV incidence among MSM aged 18−49 and discount rate. *SOC+CAB‐LA* would not be cost‐effective if HIV incidence among all MSM was halved (ICER: $13,960/YLS) or if the discount rate was 8% (ICER: $13,130/YLS).

Varying oral PrEP coverage in *SOC+CAB‐LA* (but not in SOC) also influenced ICERs. Decreasing oral PrEP coverage from 20% to 10%, corresponding to partial substitution where 10% of individuals switched from oral PrEP to CAB‐LA in *SOC+CAB‐LA*, would make *SOC+CAB‐LA* not cost‐effective (ICER: $11,270/YLS; Figure 1). Conversely, increasing oral PrEP coverage in *SOC+CAB‐LA* from 20% to 30% would preserve cost‐effectiveness (ICER: $5740/YLS). Halving HIV incidence among MSM aged 18−29 or increasing oral PrEP coverage in *SOC* (but not in *SOC+CAB‐*LA) from 20% to 30% (entailing 10% oral‐to‐LA substitution in *SOC+CAB‐LA*) would render *SOC+CAB‐LA* not cost‐effective.

*SOC+CAB‐LA* would remain cost‐effective in other scenarios, including restricting PrEP to those <30 years, varying CAB‐LA effectiveness from 85% to 96% (reflecting differences in adherence/persistence with LA PrEP relative to oral PrEP) and CAB‐LA coverage from 5% to 40% while holding oral PrEP coverage in *SOC+CAB‐LA* at 20% (i.e. no partial substitution). Varying prevalence of resistance had little impact on cost‐effectiveness. Results for TGW were qualitatively similar with lower ICERs. For MSM and TGW, *SOC+LEN‐LA* would result in slightly lower ICERs than *SOC+CAB‐LA* (Figure 1 and Supporting Information Figure A2).

### 3.3 Multiway Sensitivity Analysis Incorporating Transmission Benefits

As HIV incidence decreases, LA PrEP would need to cost less to remain cost‐effective compared to *SOC* (Supporting Information Figure A3). If HIV incidence among all MSM was halved, the maximum LA PrEP cost would be $380/year for CAB‐LA or $410/year for LEN‐LA. If HIV incidence among all MSM was doubled, then *SOC+LA* would be cost‐effective at maximum cost of $1180/year CAB‐LA or $1240/year LEN‐LA.

**FIGURE 2:**
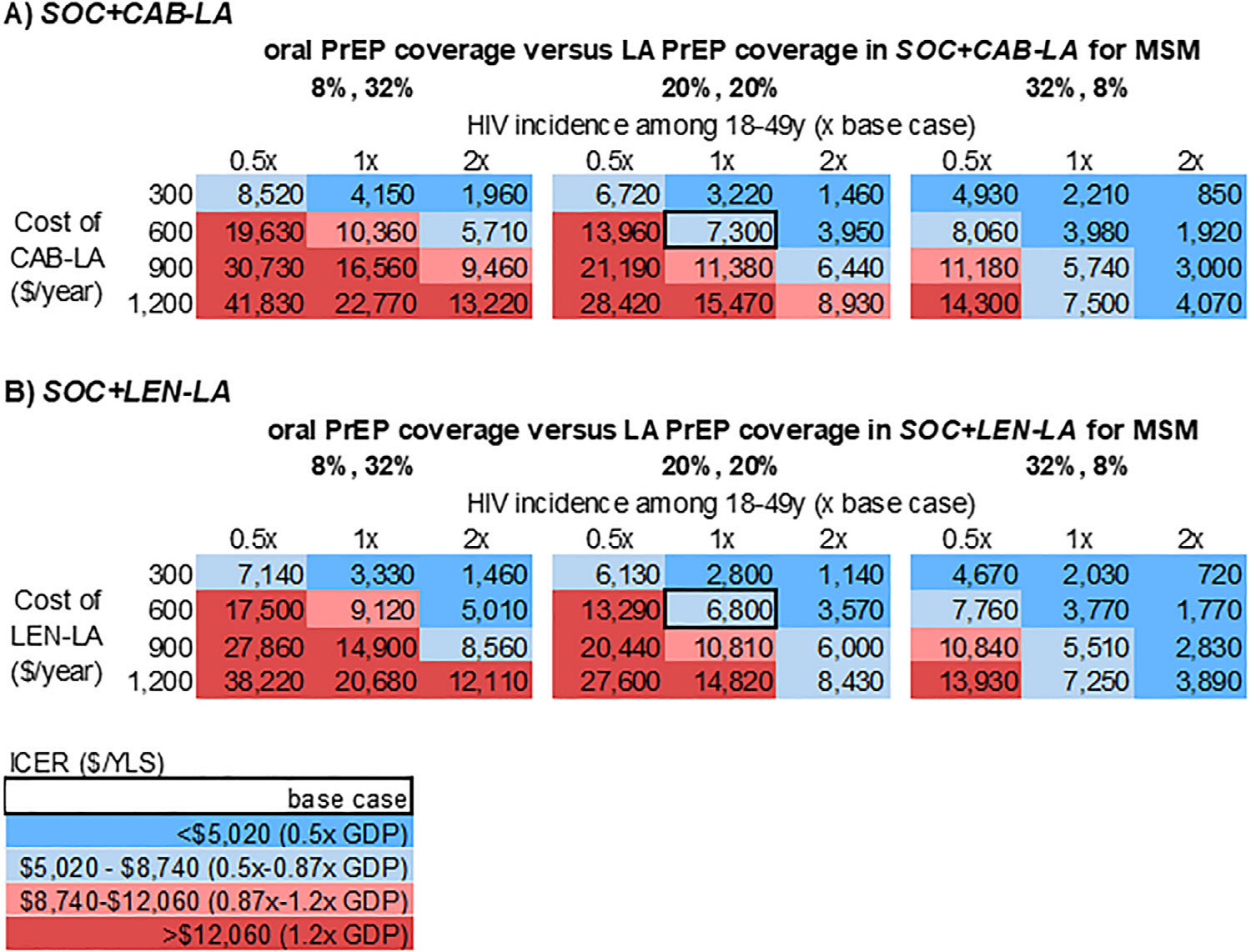
Multiway sensitivity analyses of LA PrEP cost‐effectiveness for MSM in Brazil at varying levels of HIV incidence and LA PrEP costs, for three distributions of PrEP coverage in the *SOC+LA* strategy with 40% total PrEP coverage. This figure presents a multiway sensitivity analysis on the ICER ($/YLS) of *SOC+CAB‐LA* versus *SOC* (panel A) and *SOC+LEN‐LA* (panel B) versus *SOC* when varying three parameters simultaneously: LA PrEP cost, HIV incidence among MSM 18−49 years and oral versus LA coverage in *SOC+LA*. The overall PrEP coverage in *SOC+LA* was held constant at 40%. From left to right, each of the three matrices represents a different ratio of oral to LA PrEP coverage in *SOC+LA*: [1:4], [1:1] and [4:1]. Within each matrix, the horizontal axis shows three levels of HIV incidence among MSM 18−49 years: 0.5x, 1x and 2x base case values (3.37%/year [18−29 years]/1.11%/year [30−49 years]). The vertical axis shows four annual costs of LA PrEP ($/year): 300, 600, 900 and 1200. Each cell in the matrices shows the ICER for a scenario with the specified PrEP distribution in *SOC+LA*, HIV incidence for MSM 18−49 years and LA PrEP cost. Different shades of blue represent scenarios where *SOC+LA* would be cost‐effective compared to *SOC*, with darker blue corresponding to ICERs below 0.5x GDP, or $5020/YLS, and light blue corresponding to ICERs between 0.5x GDP and the willingness‐to‐pay threshold of 0.87x GDP, or $8740/YLS. The light and darker red cells represent scenarios where *SOC+LA* would not be cost‐effective compared to SOC, with light red corresponding to ICERs between 0.87x GDP and 1.2x GDP, or $8740−$12,060/YLS, and darker red corresponding to ICERs above 1.2x GDP, or $12,060/YLS. Abbreviations: CAB‐LA, cabotegravir long‐acting; GDP, gross domestic product; ICER, incremental cost‐effectiveness ratio; LA PrEP, long‐acting pre‐exposure prophylaxis; LEN‐LA, lenacapavir long‐acting; MSM, men who have sex with men; y, year; YLS, years of life saved.

**FIGURE 3:**
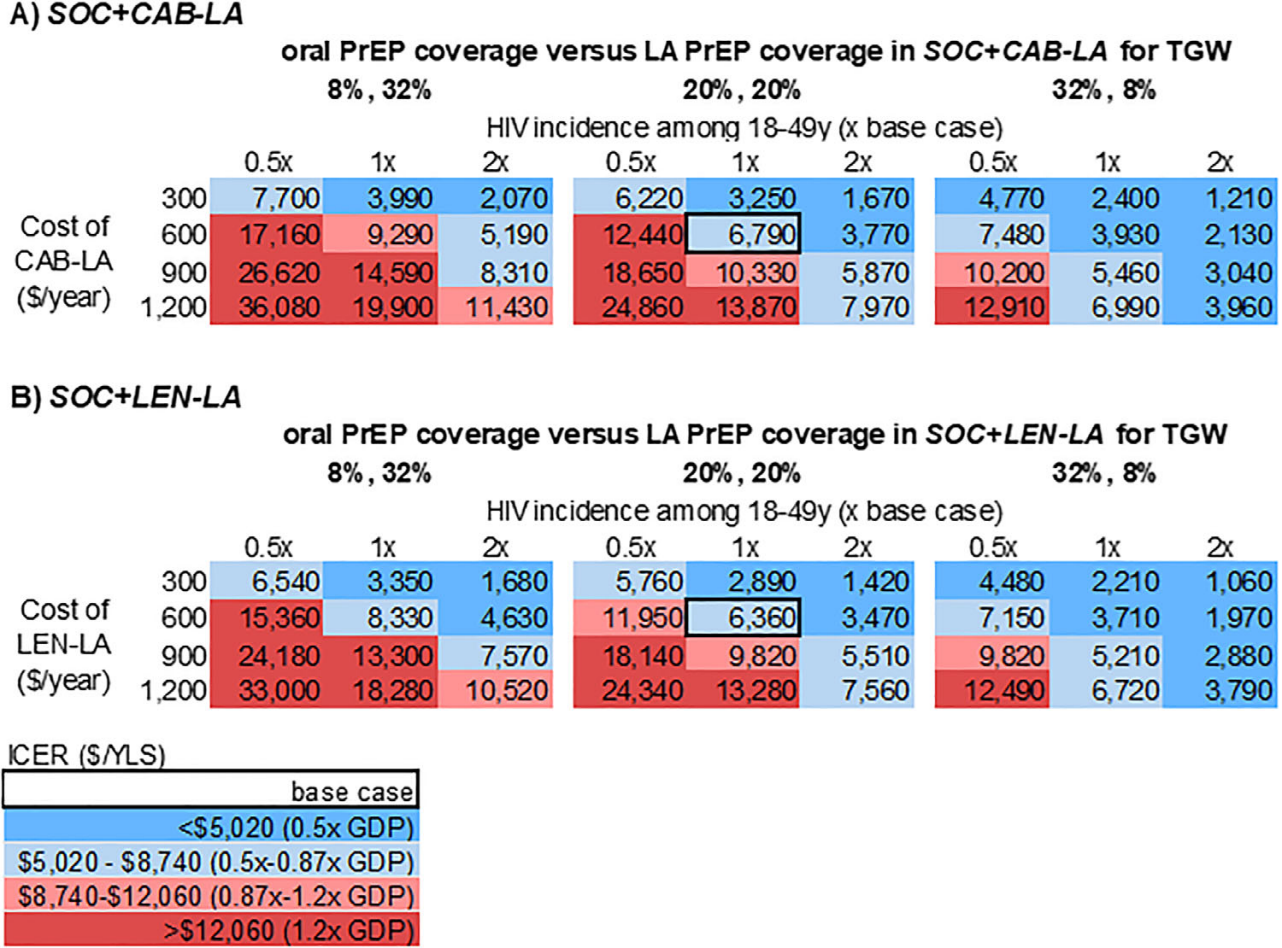
Multiway sensitivity analyses of LA PrEP cost‐effectiveness for TGW in Brazil at varying levels of HIV incidence and LA PrEP costs, for three distributions of PrEP coverage in the *SOC+LA* strategy with 40% total PrEP coverage. This figure presents a multiway sensitivity analysis on the ICER ($/YLS) of *SOC+CAB‐LA* versus *SOC* (panel A) and *SOC+LEN‐LA* (panel B) versus *SOC* when varying four parameters simultaneously: LA PrEP cost, HIV incidence among TGW 18−49 years, overall PrEP coverage and oral versus LA coverage in *SOC+LA*. From left to right, each of the three matrices represents a different ratio of oral to LA PrEP coverage in *SOC+LA*: [1:4], [1:1] and [4:1]. Within each matrix, the horizontal shows three levels of HIV incidence among TGW 18−49 years: 0.5x, 1x and 2x base case values (5.1%/year [18−29 years]/1.7%/year [30−49 years]). The vertical shows four annual costs of LA‐PrEP ($/year): 300, 600, 900 and 1200. Each cell in the matrices shows the ICER for a scenario with the specified PrEP distribution in *SOC+LA*, HIV incidence for TGW 18−49 years and LA PrEP cost. Different shades of blue represent scenarios where *SOC+LA* would be cost‐effective compared to *SOC*, with dark blue corresponding to ICERs below 0.5x GDP, or $5020/YLS, and light blue corresponding to ICERs between 0.5x GDP and the willingness‐to‐pay threshold of 0.87x GDP, or $8740/YLS. The light and darker red cells represent scenarios where *SOC+LA* would not be cost‐effective compared to SOC, with light red corresponding to ICERs between 0.87x GDP and 1.2x GDP, or $8740−$12,060/YLS, and darker red corresponding to ICERs above 1.2x GDP, or $12,060/YLS. Abbreviations: CAB‐LA, cabotegravir long‐acting; GDP, gross domestic product; ICER, incremental cost‐effectiveness ratio; LA PrEP, long‐acting pre‐exposure prophylaxis; LEN‐LA, lenacapavir long‐acting; TGW, transgender women; YLS, years of life saved.

The ICERs of *SOC+LA* versus *SOC* would change as overall PrEP coverage and the ratio of oral PrEP versus LA PrEP coverage change in *SOC+LA* (Figures 2, 3 and Supporting Information Figures A4 and A5). Doubling HIV incidence among all MSM and changing coverage of oral versus LA PrEP to 32%:8% (base case: 20%:20%) would decrease ICER (<$4070/YLS with *SOC+CAB‐LA* or <$3890/YLS with *SOC+LEN‐LA*) at LA PrEP cost <$1200/year (Figure 2). Under partial substitution scenarios, where coverage of oral versus LA PrEP was 8%:32% (12% of MSM on oral PrEP switched to LA PrEP and additionally 20% of MSM not on PrEP started LA PrEP), the value of *SOC+LA* would decrease relative to the base case with additive coverage (20%:20%, Figure 2). If the introduction of LA PrEP increased overall PrEP coverage to only 30% (base case: 40%), then the value of *SOC+LA* would decrease, especially in scenarios where incidence was low (Supporting Information Figure A4). When HIV incidence among all MSM was halved and coverage of oral versus LA PrEP was 6%:24% in *SOC+LA*, the ICER of *SOC+LA* versus *SOC* would be >$21,020//YLS at LA PrEP cost ≥$600/year, exceeding the willingness‐to‐pay threshold (Supporting Information Figure A4). Results for TGW are similar, with lower ICERs in all corresponding cases (Figure 3 and Supporting Information Figure A5).

### 3.4 Scenario Analysis Incorporating Transmission Benefits

Under the more pessimistic scenario analysis with partial substitution coverage, clinical benefit and cost‐effectiveness of *SOC+LA* over *SOC* were attenuated compared to the base case (Supporting Information Table A2).

## 4 Discussion

We evaluated the clinical impact, cost and cost‐effectiveness of LA PrEP with either CAB‐LA or LEN‐LA to prevent HIV acquisition among MSM and TGW in Brazil. We found that providing options of both oral and LA PrEP to MSM and TGW would markedly increase LE and reduce 10‐year and lifetime HIV risk. Compared to standard‐of‐care oral PrEP at current coverage, this strategy would be cost‐effective if CAB‐LA cost less than $710/year. Cost thresholds for LEN‐LA would be slightly higher at $740/year given the higher effectiveness of LEN‐LA. In more pessimistic scenarios, when indirect transmission benefits were excluded from ICER calculations, cost thresholds would be lower, at $410/year for CAB‐LA and $430/year for LEN‐LA.

Introducing LA PrEP to the Brazilian public health system would lead to tangible additional clinical improvements—life‐years gained and decreased HIV risk—compared to standard‐of‐care. However, these benefits do not necessarily justify a large cost differential between LA PrEP and oral PrEP, due to the wide availability of affordable and effective oral PrEP and antiretroviral treatment. At a suggested cost of $1440/year [25], implementing LA PrEP among MSM and TGW in Brazil, an upper middle‐income country, either with CAB‐LA or LEN‐LA, would not provide good value compared to the existing standard‐of‐care, with an ICER above $10,360/YLS (>1x Brazil GDP per capita).

Our findings also suggest that LA PrEP would have greater value in TGW than MSM due to higher HIV incidence in TGW. Since the number of TGW in Brazil is substantially smaller than the number of MSM, LA‐PrEP in TGW would also be more affordable from a budgetary perspective. Decisions about equity among key populations, however, should also reflect broader societal norms and values [68].

The projected clinical improvements introduced by adding LA PrEP to standard‐of‐care were mainly due to increased overall coverage of PrEP regardless of modality. Initial results from the ImPrEP CAB Brasil implementation study showed that given a choice between oral and CAB‐LA, 83% of participants chose CAB‐LA (17% oral PrEP), and 95% of participants using CAB‐LA were covered by study drug during follow‐up (58% for oral PrEP). Though how well introducing LA PrEP will promote engagement with any PrEP product in non‐study settings is unknown, results are promising [67, 69]. Our results show that the impact of adding LA PrEP as an option on overall PrEP coverage was important. If introducing LA PrEP increased overall PrEP coverage mainly by increasing oral PrEP coverage, this would increase the value of a combined strategy providing LA PrEP and oral PrEP. In contrast, the value of including LA PrEP would decrease if adding it caused some oral PrEP users to switch to LA PrEP. Despite the potential impact and value of LA PrEP, challenges include potential development of drug resistance [55, 58]. We found, however, that even when assuming that 50% of those failing CAB‐LA would develop INSTI‐based resistance, the impact on LA PrEP value at the population level would be small, since the number of people infected while on PrEP is low.

Others have evaluated the value of LA PrEP in other settings and arrived at conclusions similar to ours [30, 59, 70, 71, 72]. In the United States, it was found that CAB‐LA would be cost‐effective for MSM and TGW by US standards compared to generic oral PrEP if priced below $4100/year (oral PrEP: $400/year); this is well below the current CAB‐LA cost of $22,200/year [30]. Another study of CAB‐LA for MSM at increased vulnerability to HIV acquisition in three countries found that although CAB‐LA would offer substantial clinical benefit, CAB‐LA cost in all three regions would need to be reduced by 50%–90% to be cost‐effective compared to oral PrEP [70]. Our results reinforce the dependence of CAB‐LA value on affordable pricing and prioritization in populations with very high HIV incidence. As newer PrEP regimens become available, it will be important to compare their effectiveness, cost and acceptability with that of CAB‐LA and LEN‐LA [73].

This analysis has several limitations. First, there is not yet a cost for CAB or LEN in Brazil. For LEN, the stated target price is $40/year, or $20/dose in LMICs [74]. It is not clear, however, whether this will be achieved and available in Brazil. We examined a wide range of potential costs for CAB and LEN to understand the impact of this uncertainty. While our results can inform the potential impact and value of LA PrEP at different costs, the results are meant to inform price discussions and the potential for countries to adopt a novel intervention rather than a specific price point for CAB‐LA or LEN‐LA.

A second limitation is that we only incorporated the benefit of averting one generation of primary HIV transmissions. Including the benefit of averting additional generations of transmissions would increase LA PrEP value, although as incidence decreases in the future, PrEP would be of less value. Third, although we did not model differences in individual behaviours of discontinuing or restarting PrEP, our modelling strategy allowed us to capture PrEP effectiveness on an average level as well as the superiority of LA PrEP in promoting longer durations of effective drug concentrations via simplifying adherence compared to oral PrEP. Fourth, due to uncertainty around how LA PrEP would promote PrEP engagement and persistence in clinical settings, we simulated a base case with LA PrEP coverage additive to oral PrEP coverage and a more pessimistic scenario analysis with partial substitution coverage. This showed how much outcomes depended on whether LA PrEP expands total PrEP coverage or mainly redistributes existing coverage across modalities. This distinction had an impact on cost‐effectiveness results. We varied both LA PrEP and oral PrEP coverage widely, however, and found that key conclusions remain unchanged; LA PrEP would be most cost‐effective if its introduction would also increase oral PrEP coverage. Fifth, we did not consider HIV incidence over age 50, though this is low in Brazil [43]. Lastly, a time‐phased trajectory in which coverage increases progressively from current levels would provide a more realistic representation of its implementation, with LA‐PrEP cost‐effectiveness emerging after sustained increases in total coverage. Implementation of LA PrEP is likely to occur in a phased manner, beginning in larger cities with higher HIV incidence. Such a targeted rollout would enhance the impact and cost‐effectiveness of LA PrEP, with broader expansion occurring as programmatic capacity expands. Although our model does not explicitly simulate phased implementation, our findings can inform prioritization strategies for incremental adoption.

The cost‐effectiveness thresholds identified in this analysis are based on Brazil's epidemiology, health system organization and cost structure, and willingness‐to‐pay threshold. They should not be interpreted as regionwide benchmarks, since cost‐effectiveness thresholds in Brazil may not be feasible in countries with smaller health budgets. Furthermore, our results do not indicate that LA PrEP would necessarily be affordable for Brazil's health system; affordability is a separate component of decision‐making. Budget constraints, competing health priorities and procurement conditions could restrict the ability to adopt LA PrEP even when cost‐effectiveness criteria are met. Indeed, affordability of LA PrEP in Latin America may benefit from procurement mechanisms beyond national purchasing. Regional initiatives, such as PAHO's Strategic Fund, MERCOSUR's health integration initiative or other joint negotiation platforms could facilitate access to lower costs. The cost thresholds identified here may be useful in informing ongoing regional dialogues on PrEP procurement.

## 5 Conclusions

In conclusion, we found that introducing LA PrEP to MSM/TGW in Brazil in addition to oral PrEP could markedly improve long‐term clinical outcomes. LA PrEP would provide greatest value if prioritized in populations at highest HIV incidence. These products would be cost‐effective in Brazil if annual costs were approximately $700 and the impact and value in Brazil and similar settings will be contingent on having prices substantially lower than those in high‐income countries. Ongoing negotiations to bring down the cost of LA PrEP in upper‐middle‐income countries with high HIV burden are critical to realize the potential of these novel agents to decrease incidence and ensure further progress in addressing the HIV epidemic.

## Author Contributions

All authors contributed substantively to this manuscript in the following ways: study design (WC, KAF, BG, PML, VGV, JAS), data analysis (WC, PML, AS), interpretation of results (WC, CMD, KAF, PML, AS, JAS), drafting the manuscript (WC, KAF, PML, AS, JAS), critical revision of the manuscript (all authors) and final approval of submitted version (all authors).

## Funding

This work was supported by the National Institute of Allergy and Infectious Diseases (R37 058736 [KAF]) and the Eunice Kennedy Shriver National Institute of Child Health and Human Development (R01 HD111355 [AMN]).

## Conflicts of Interest

The authors declare no conflicts of interest.

## Disclaimer

The content is solely the responsibility of the authors and does not necessarily represent the official views of the US National Institutes of Health.

## Ethics Statement

This study was approved by the Mass General Brigham Human Research Committee. No patient‐level data were included in this modelling study, so no consent was required.

## Supporting information




**Supporting File 1**: jia270116‐sup‐0001‐figureS1.png

**Supporting File 2**: jia270116‐sup‐0002‐figureS2.png

**Supporting File 3**: jia270116‐sup‐0003‐figureS3.png

**Supporting File 4**: jia270116‐sup‐0004‐figureS4.png

**Supporting File 5**: jia270116‐sup‐0005‐figureS5.png

**Table A1**: Additional model inputs for a cost‐effectiveness analysis of LA PrEP cost‐effectiveness of MSM and TGW.

**Table A2**: Results of an analysis of LA PrEP cost‐effectiveness for MSM and TGW in Brazil under a pessimistic scenario: partial substitution coverage (15% oral PrEP and 15% LA PrEP for a total coverage of 30%)

**Supporting File 6**: jia270116‐sup‐0008‐SuppMat.docx

## Data Availability

All data used in this study are available in the manuscript, appendix, or on the CEPAC website (https://mpec.massgeneral.org/cepac-model/).
