# Supplementary figures and images for: Long‐Acting PrEP for People With High Vulnerability to HIV Acquisition in Brazil: A Cost‐Effectiveness Analysis
Source: J Int AIDS Soc. 2026 May 14;29(5):e70116. doi: 10.1002/jia2.70116 (PMC13176634; doi:10.1002/jia2.70116)

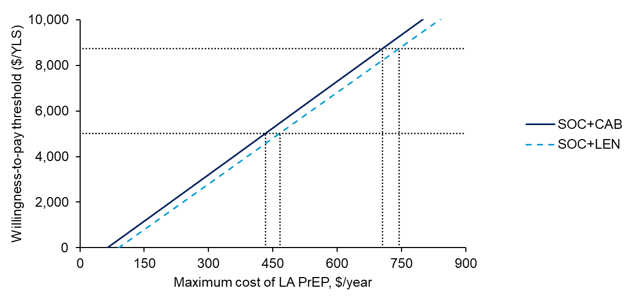

Supplement: Supplementary file 1 — Supporting File 1: jia270116‐sup‐0001‐figureS1.png [file JIA2-29-e70116-s004.png]

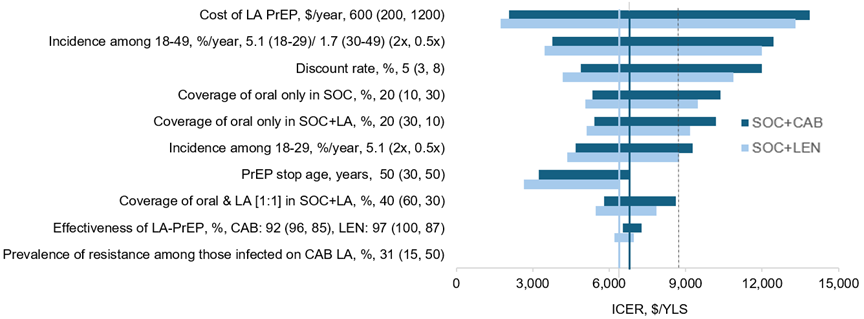

Supplement: Supplementary file 2 — Supporting File 2: jia270116‐sup‐0002‐figureS2.png [file JIA2-29-e70116-s008.png]

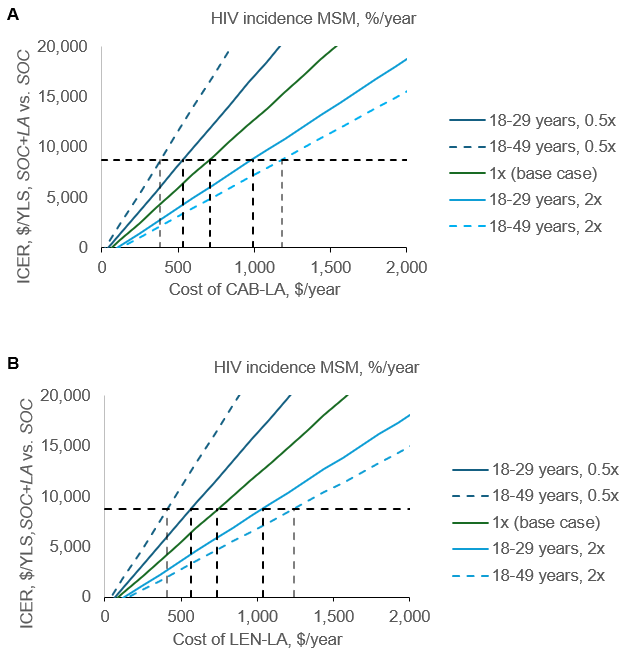

Supplement: Supplementary file 3 — Supporting File 3: jia270116‐sup‐0003‐figureS3.png [file JIA2-29-e70116-s006.png]

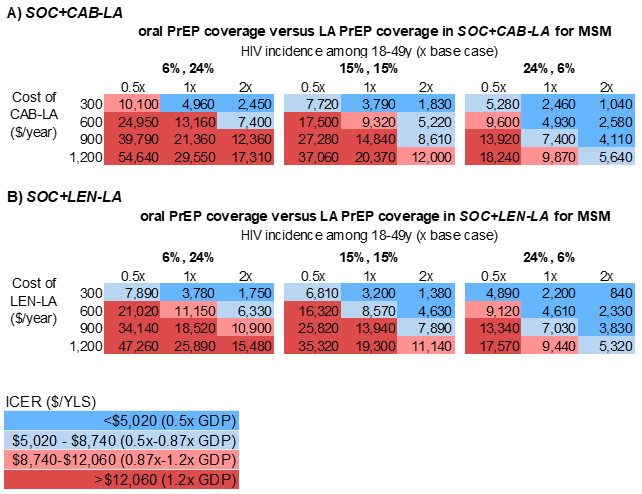

Supplement: Supplementary file 4 — Supporting File 4: jia270116‐sup‐0004‐figureS4.png [file JIA2-29-e70116-s002.png]

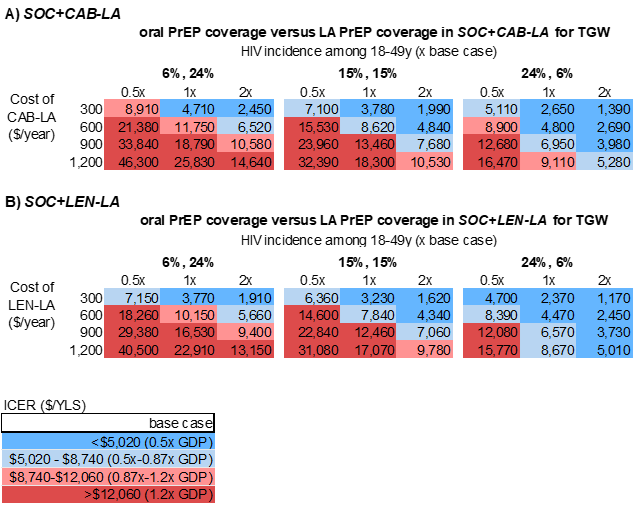

Supplement: Supplementary file 5 — Supporting File 5: jia270116‐sup‐0005‐figureS5.png [file JIA2-29-e70116-s007.png]
