# Supplementary material for: Long‐Acting PrEP for People With High Vulnerability to HIV Acquisition in Brazil: A Cost‐Effectiveness Analysis
Source: J Int AIDS Soc. 2026 May 14;29(5):e70116. doi: 10.1002/jia2.70116 (PMC13176634; doi:10.1002/jia2.70116)
Supplement: Supplementary file 6 — Table A1: Additional model inputs for a cost‐effectiveness analysis of LA PrEP cost‐effectiveness of MSM and TGW. [file JIA2-29-e70116-s001.docx]

**Supporting information Table A1.** Additional model inputs for a cost-effectiveness analysis of LA PrEP cost-effectiveness of MSM and TGW.

|  | **Value** | **Reference** |
| --- | --- | --- |
| **HIV care** |  |  |
| Increase in CD4 count after 48 weeks on suppressive ART, cells/µL, mean (SD) |  | [22] |
| First-line (TLD) |  |  |
| First two months | 80.4 (30) |  |
| After Month 2 | 4.02 (1.5) |  |
| Second-line (DRV/r + 2NRTIs) |  |  |
| First 2 months | 83.2 (38.175) |  |
| After month 2 | 4.02 (1.5) |  |

| Suppression at 12 months, % |  | Der. from [23,24] |
| --- | --- | --- |
| First-line (TLD) | 88 |  |
| Second-line (DRV/r+2NRTIs) | 73 |  |
| Rate of virologic failure, incidence/100 person-months |  | Der. from [25,26] |
| First-line (TLD) | 0.35 |  |
| Second-line (DRV/r+2NRTIs) | 0.93 |  |
| Rate of disengagement from HIV care/ 100 person-years |  | INI^†^ |
| MSM | 11.6 |  |
| TGW | 27.3 |  |

**Supporting information Table A1, continued.** Additional model inputs for a cost-effectiveness analysis of LA PrEP cost-effectiveness of MSM and TGW.

|  | | | **Value** | **Reference** |
| --- | --- | --- | --- | --- |
| **MSM HIV care continuum in 2021** | | | |  |
| MSM with HIV, N | 476,530 | | Derived from [4–9] |  |
| On ART among MSM with HIV, % | 64% | |  |  |
| MSM without HIV, n | 1,357,050 | |  |  |
| New infections, n | 29,750 | | Derived from [4–10] |  |
| **TGW HIV care continuum in 2021** | | | |  |
| TGW with HIV, n | 384,950 | | Derived from [4,5,12–15,27] |  |
| Percentage on ART among TGW with HIV, % | 59% | |  |  |
| TGW without HIV, n | 679,960 | |  |  |
| New infections, n | 21,480 | | Derived from [4,5,10,12–16,27] |  |
| **Cost** | | | |  |
| HIV viral load test, $/test | 12.27 | | [18,21] |  |
| CD4 count test, $/test | 10.84 | | [18,21] |  |

**Supporting information Table A1, continued.** Additional model inputs for a cost-effectiveness analysis of LA PrEP cost-effectiveness of MSM and TGW.

|  | **Value** | **Reference** |  |
| --- | --- | --- | --- |
| Acute | 100.72 | [1–10] |  |
| >100,000 copies/mL | 14.54 |  |  |
| 10,000-100,000 copies/mL |  | 13.07 |  |
| 3,000-10,000 copies/mL | 6.71 |  |  |
| 20-3,000 copies/mL |  | 3.32 |  |
| ≤20 copies/ML | 0 |  |  |
| Acute |  | 8.03 | [1–10,17] |
| >100,000 copies/mL | 1.16 |  |  |
| 10,000-100,000 copies/mL |  | 1.04 |  |
| 3,000-10,000 copies/mL | 0.53 |  |  |
| 20-3,000 copies/mL |  | 0.26 |  |
| ≤20 copies/ML | 0 |  |  |

| Abbreviations: ART, antiretroviral therapy; DRV/r, darunavir/ritonavir; LA PrEP, long-acting pre-exposure prophylaxis; MSM, men who have sex with men; NRTIs, nucleoside reverse transcriptase inhibitors; SD, standard deviation, TGW, transgender women; TLD, tenofovir disoproxil, lamivudine, and dolutegravir |
| --- |
