# Supplementary material for: Long‐Acting PrEP for People With High Vulnerability to HIV Acquisition in Brazil: A Cost‐Effectiveness Analysis
Source: J Int AIDS Soc. 2026 May 14;29(5):e70116. doi: 10.1002/jia2.70116 (PMC13176634; doi:10.1002/jia2.70116)
Supplement: Supplementary file 7 — Table A2: Results of an analysis of LA PrEP cost‐effectiveness for MSM and TGW in Brazil under a pessimistic scenario: partial substitution coverage (15% oral PrEP and 15% LA PrEP for a total coverage of 30%) [file JIA2-29-e70116-s005.docx]

**Supporting information Table A2.** Results of an analysis of LA PrEP cost-effectiveness for MSM and TGW in Brazil under a pessimistic scenario: partial substitution coverage (15% oral PrEP and 15% LA PrEP for a total coverage of 30%)

| Strategy | Undisc.  LE, y | Disc.  LE, y | 10-year  infection risk, % | Lifetime  infection risk^‡^, % | Undisc. costs, $ | Disc.  costs, $ | ICER^†^, $/YLS |
| --- | --- | --- | --- | --- | --- | --- | --- |
| **MSM** | | | | | | | |
| LA PrEP option = cabotegravir |  |  |  |  |  |  |  |
| *SOC* | 39.0 | 16.2 | 14.0 | 21.4 | 2,800 | 1,060 | - |
| *SOC+CAB-LA* | 39.2 | 16.3 | 12.3 | 18.9 | 4,210 | 1,990 | 9,320 |
| LA PrEP option = lenacapavir |  |  |  |  |  |  |  |
| *SOC* | 39.0 | 16.2 | 14.0 | 21.4 | 2,800 | 1,060 | - |
| *SOC+LEN-LA* | 39.2 | 16.3 | 12.2 | 18.7 | 4,130 | 1,950 | 8,570 |
| **TGW** | | | | | | | |
| LA PrEP option = cabotegravir |  |  |  |  |  |  |  |
| *SOC* | 36.0 | 15.7 | 20.0 | 29.5 | 2,660 | 1,080 | - |
| *SOC+CAB-LA* | 36.5 | 15.8 | 17.7 | 26.1 | 4,050 | 1,990 | 8,620 |
| LA PrEP option = lenacapavir |  |  |  |  |  |  |  |
| *SOC* | 36.0 | 15.7 | 20.0 | 29.5 | 2,660 | 1,080 | - |
| *SOC+LEN-LA* | 36.5 | 15.8 | 17.5 | 25.8 | 3,980 | 1,950 | 7,840 |
| †IThis is the discounted ICER of SOC+LA compared to SOC, where the cost of CAB-LA or LEN-LA is $600/year, adjusted to include the benefit of reduced primary transmissions. Without adjusting for transmissions, the discounted ICERs would be $16,210/YLS (MSM) and $9,240/YLS (TGW) for SOC+CAB-LA versus SOC, or $15,470/YLS (MSM) and $8,430/YLS (TGW) for SOC+LEN-LA versus SOC.  ‡We did not model HIV incidence above age 50.  Abbreviations: Disc., discounted; ICER, incremental cost-effectiveness ratio; LE, life expectancy; LA PrEP, long-acting pre-exposure prophylaxis; MSM, men who have sex with men; SOC, standard-of-care using oral PrEP at 20% coverage; SOC+LA, strategy providing both oral PrEP (coverage 15%) and LA PrEP (coverage 15%) using CAB-LA or LEN-LA; TGW, transgender women; Undisc., undiscounted | | | | | | | |
