# Supplementary material for: Long‐Acting PrEP for People With High Vulnerability to HIV Acquisition in Brazil: A Cost‐Effectiveness Analysis
Source: J Int AIDS Soc. 2026 May 14;29(5):e70116. doi: 10.1002/jia2.70116 (PMC13176634; doi:10.1002/jia2.70116)
Supplement: Supplementary file 8 — Supporting File 6: jia270116‐sup‐0008‐SuppMat.docx [file JIA2-29-e70116-s003.docx]

**Supporting information**

**Long-acting PrEP for people with high vulnerability to HIV acquisition in Brazil: A cost-effectiveness analysis**

Wanyi Chen*

Paula M. Luz*

Anjali Srinivasan

Beatriz Grinsztejn

Justine A. Scott

Anne M. Neilan

Valdiléa G. Veloso

Caitlin M. Dugdale

Kenneth A. Freedberg

*Authors contributed equally to the manuscript

**INTRODUCTION**

In this supporting information document, we provide additional methods and results, including a scenario analysis and one-way and multiway sensitivity analyses.

**METHODS**

**CEPAC model structure**

At model start, all individuals are HIV uninfected and have monthly probabilities of acquiring HIV that vary by age and subpopulation. Upon acquiring HIV, individuals are assigned an initial CD4 count and HIV RNA level. Without effective antiretroviral therapy (ART), CD4 count declines monthly. Further, individuals face risks of developing opportunistic infections (OIs) and chronic AIDS-related mortality, stratified by CD4 count. Once diagnosed, individuals with HIV are linked to HIV care and prescribed ART. Successful ART decreases HIV RNA levels and increases CD4 count, which leads to reduced mortality from OIs or chronic AIDS. Additional details about the CEPAC model can be found at <https://mpec.massgeneral.org/cepac-model/>.

**Incorporating the benefits of averted primary HIV transmissions into ICERs**

We report ICERs that include the benefits of averted primary HIV transmissions. To implement this, we 1) project one generation of primary transmissions from the “Initial MSM” and “Initial TGW” cohorts, aged 18-49, in each year over a lifetime horizon for each modeled strategy. The difference in number of annual transmissions between *SOC* and *SOC+LA* is the number of annual transmissions averted by the introduction of LA PrEP. 2) We estimate the benefits, i.e., life-years (LYs) gained, and lifetime costs saved, by averting a single transmission each year through the following: we calculate the lifetime difference in discounted LYs and costs between a person who becomes acutely infected in that year and a person who is without HIV in that year but faced age-specific incidence thereafter. 3) We assume random sexual mixing by age, i.e., primary transmissions occur in people outside the “Initial cohorts” that have the same age distribution as the “Initial cohorts”. 4) We discount the benefits estimated in step 2 at 5%/year to obtain the present value of the LYs and costs saved by averting a single transmission in each year in the future. 5) We multiply these estimated discounted benefits in their present values by the number of annual transmissions averted from step 1, and then sum these across lifetime to determine the total LYs and lifetime costs saved by *SOC+LA*, via averting one generation of primary transmissions. 6) We include these estimated LYs gained and lifetime costs saved from averted primary transmissions in the final LYs and costs for the *SOC+LA* strategy, by adding the LYs saved and subtracting the costs saved, based on which we calculate the ICERs of *SOC+LA* versus *SOC*.

**Transmission rate calibration**

We calibrate transmission rates as follows: 1) we use published estimates of transmission rates as a starting point (**Table 1**) [1–3], varying by HIV RNA levels, with the highest transmission rates for those with acute infection and not on ART. 2) We project the total number of transmissions for 1 year (2021-2022) from three cohorts representative of the HIV care continuum of Brazil among MSM in year 2021 (**Supporting information Table A1**) [4–15]: 1) “Initial cohorts” of MSM without HIV and face age-specific incidence thereafter; 2) “On ART” cohorts who are with chronic HIV and on ART at time 0, and can disengage from/reengage with care thereafter; and 3) “Off ART” cohorts who are with chronic HIV and not linked to care at time 0, and can link to care through background testing, and face the same probabilities of care engagement/disengagement as the “On ART” cohorts. 3) We apply a uniform transmission rate multiplier to the estimated total transmissions to match the number of new infections in year 2021 as seen in the ”initial cohort” from Brazil (**Supporting information Table A1**) [4–16]. Because TGW engage primarily in receptive anal sex, which carries a 12.5 times lower per-act risk of HIV transmission than insertive anal risk, we divided the MSM transmission rate multiplier by 12.5 for TGW [12,17].

**PrEP effectiveness**

We modeled oral PrEP effectiveness as the product of efficacy (percent HIV risk reduction with ≥4 pills/week compared to no PrEP) and adherence (percentage of time an individual takes ≥4 pills/week). In the iPrEx trial, oral PrEP had an efficacy of 96% with ≥4 doses/week [42]. We use adherence of 78% for daily oral PrEP from the literature [43], for an effectiveness of 75%.

For LA PrEP, HPTN 083 showed a 66% reduction (95% CI: 38-82%) in HIV acquisition with CAB-LA compared with oral PrEP by intention-to-treat [16]. Similarly, PURPOSE 2 showed an 89% reduction (95% CI: 49-98%) in HIV acquisition with LEN-LA compared with oral PrEP by intention-to-treat [17]. Reductions in HIV incidence observed with LA PrEP in these trials were driven by a combination of improved PrEP adherence (i.e., number of days covered by therapeutic drug levels) and greater PrEP program persistence (i.e., longitudinal engagement/attendance at refill/redosing visits) with LA PrEP.

In the base case, we assumed the modeled population would have similar adherence, persistence, and biologic efficacy with LA PrEP compared to oral PrEP as observed in HPTN 083 and PURPOSE 2 and applied the HIV risk reductions in those trials with LA PrEP vs. oral PrEP to model LA PrEP effectiveness. This resulted in an estimated LA PrEP effectiveness of 92% (95%CI 85-96%) with CAB-LA from the 66% (95% CI: 38-82%) relative risk reduction in HIV acquisition [16] in HPTN 083 and 97% (95% CI 87-100%) effectiveness with LEN-LA from the 89% (95% CI: 49-98%) relative risk reduction in PURPOSE 2 [17]. We varied effectiveness assumptions in sensitivity analyses to reflect clinical practice where people may have lower PrEP adherence or persistence than observed in trials.

**Costs**

We derived provider time costs by multiplying provider time from a PrEP demonstration project time-and-motion study by unit provider salary in Brazil [18–21], which leads to $5.50/initiation and $3.00/follow-up visit related to the oral PrEP program.

**RESULTS**

**One-way sensitivity analyses**

Among TGW, LA PrEP cost was most influential on the cost-effectiveness of *SOC+LA* versus *SOC* (**Supporting information Figure A2**). A decrease to $200/year would yield an ICER of $2,070/YLS with CAB-LA, and $1,740/YLS with LEN-LA. When cost increased to $1,200/year, *SOC+ LA* would not be cost-effective given a willingness-to-pay threshold of $8,740/YLS (ICER: $13,870/YLS with SOC+CAB-LA, or $13,290/YLS with SOC+LEN-LA). The next most influential parameter was HIV incidence among TGW aged 18-49. *SOC+CAB-LA* would not be cost-effective if HIV incidence among all TGW was halved (2.5%/year age 18-29 years and 0.8%/year age 30-49 years [base case: 5.1%/year and 1.7%/year]), with an ICER of $12,440/YLS with CAB-LA or $11,960 with LEN-LA. The discount rate was also influential, with a discount rate of 8% resulting in ICERs that exceed the willingness-to-pay threshold. Other parameters that were found to be influential included coverage of oral PrEP in *SOC* and *SOC+LA* (varied individually but not simultaneously), overall PrEP coverage in *SOC+LA* (with ratio of oral vs. LA coverage held constant at 1:1), PrEP stop age (for both oral and LA), and effectiveness of LA PrEP. Like the results for MSM, we did not find varying prevalence of resistance while failing CAB-LA to be influential on the ICER of *SOC+CAB-LA* versus *SOC* for TGW.

**Multiway sensitivity analyses**

Two-way sensitivity analyses focused on HIV incidence and LA PrEP cost. As HIV incidence decreases, LA PrEP would need to cost less to remain cost-effective compared to *SOC* (**Supporting information Figure A3**).

The comparative value of SOC+LA versus SOC would change as the overall PrEP coverage and the ratio of oral PrEP versus LA PrEP coverage change in SOC+LA (**Supporting information Figure A4**).

Similar to MSM, for TGW, when the overall PrEP coverage and the ratio of oral PrEP versus LA PrEP coverage were varied simultaneously in addition to HIV incidence and LA PrEP cost, the comparative value of *SOC+LA* versus *SOC* would further change (**Supporting information Figure A5**). If the overall PrEP coverage in *SOC+LA* only increased slightly compared to oral PrEP coverage in *SOC*, then the value of *SOC+LA* would decrease. For example, when HIV incidence among all TGW was halved and when coverage of oral versus LA PrEP was 6%:24% in *SOC+LA* (overall coverage 30%, effectively switching 14% of oral PrEP users and 10% of PrEP non-users in *SOC* to LA PrEP), then the ICER of *SOC+LA* versus *SOC* would be over $21,380/YLS with *SOC+CAB-LA* or over $18,260/YLS with *SOC+LEN-LA* at LA PrEP cost ≥$600/year (base case: $6,790/YLS with *SOC+CAB-LA* or $6,360/YLS with *SOC+LEN-LA* at LA PrEP $600/year). On the contrary, if introducing LA PrEP increased overall PrEP coverage mainly through bringing in more oral PrEP users, then the value of *SOC+LA* would improve. For instance, if HIV incidence among all TGW was doubled and coverage of oral PrEP versus LA PrEP was 32%:8% (base case: 20%:20%), then the ICER would be ≤$3,960/YLS with *SOC+CAB-LA* or ≤$3,790 with *SOC+LEN-LA* if LA PrEP cost no more than $1,200/year.

**Number needed to treat calculation**

For each PrEP modality (oral PrEP, CAB-LA, and LEN-LA), we calculated the number needed to treat (NNT) to prevent one primary infection over a 10-year time horizon, using a counterfactual no PrEP scenario as the reference comparator. This approach allows NNT to reflect the absolute preventive effect of each modality over a defined time horizon. Specifically, for each PrEP modality we conducted two model runs: 1) no PrEP (0% coverage) and 2) universal PrEP use (100% coverage) with the corresponding PrEP modality. We then calculated the absolute risk reduction (ARR) in cumulative 10-year HIV incidence between these two runs. NNT was calculated as the inverse of ARR: NNT = 1/ARR. The NNT thus represents the number of individuals who would need to receive the specified PrEP modality for 10 years to prevent one primary HIV infection compared with no PrEP.

**REFERENCES**

1. Attia S, Egger M, Müller M, Zwahlen M, Low N. Sexual transmission of HIV according to viral load and antiretroviral therapy: systematic review and meta-analysis. AIDS. 2009 July 17;23(11):1397–404.

2. Eisinger RW, Dieffenbach CW, Fauci AS. HIV viral load and transmissibility of HIV infection: undetectable equals untransmittable. JAMA. 2019 Feb 5;321(5):451–2.

3. Bellan SE, Dushoff J, Galvani AP, Meyers LA. Reassessment of HIV-1 acute phase infectivity: accounting for heterogeneity and study design with simulated cohorts. PLoS Med. 2015 Mar;12(3):e1001801.

4. Division UNP. By location | pivot table | data portal [Internet]. Population Division Data Portal. [cited 2025 Aug 19]. Available from: https://population.un.org/dataportal/data/indicators/46/locations/76/start/2023/end/2024/table/pivotbylocation?df=b911cc37-d409-496d-9c18-54e3adc2bc84

5. Pesquisa Nacional de Saúde [Internet]. The Brazilian Institute of Geography and Statistics or Instituto Brasileiro de Geografia e Estatística. [cited 2025 Aug 19]. Available from: https://biblioteca.ibge.gov.br/visualizacao/livros/liv101800.pdf

6. Silva KRO, Ferreira RC, Coelho LE, Veloso VG, Grinsztejn B, Torres TS, et al. Knowledge of HIV transmission, prevention strategies and U = U among adult sexual and gender minorities in Brazil. J Int AIDS Soc. 2024 Feb;27(2):e26220.

7. Jalil CM, Torres TS, Jalil EM, Scarparo RO, Nazareth DC, Ribeiro GT, et al. HIV prevention and treatment cascades among young Brazilian MSM: results from the Conectad@s project. Prevenção e tratamento de HIV em cascatas entre jovens HSH brasileiros: Resultados do Projeto Coectad@S [Internet]. 2023 [cited 2024 Sept 3]; Available from: https://www.arca.fiocruz.br/handle/icict/60907

8. Survey of knowledge, attitudes and practices in the brazilian population - PCAP 2013 | department of chronic conditions and sexually transmitted infections [Internet]. [cited 2025 Aug 19]. Available from: http://antigo.aids.gov.br/pt-br/pub/2016/pesquisa-de-conhecimentos-atitudes-e-praticas-na-populacao-brasileira-pcap-2013

9. Relatório de Monitoramento Clinico do HIV 2022 — Ministério da Saúde [Internet]. [cited 2025 Aug 19]. Available from: https://www.gov.br/saude/pt-br/centrais-de-conteudo/publicacoes/svsa/aids/relatorio-de-monitoramento-clinico-do-hiv-2022.pdf/view

10. Torres TS, Teixeira SLM, Hoagland B, Konda KA, Derrico M, Moreira RI, et al. Recent HIV infection and annualized HIV incidence rates among sexual and gender minorities in Brazil and Peru (ImPrEP seroincidence study): a cross-sectional, multicenter study. Lancet Reg Health Am. 2023 Dec;28:100642.

11. IBGE vai estimar tamanho da população trans e travesti no Brasil | Radioagência Nacional [Internet]. [cited 2025 Aug 19]. Available from: https://agenciabrasil.ebc.com.br/radioagencia-nacional/direitos-humanos/audio/2023-10/ibge-vai-estimar-tamanho-da-populacao-trans-e-travesti-no-brasil

12. Grinsztejn B, Jalil EM, Monteiro L, Velasque L, Moreira RI, Garcia ACF, et al. Unveiling HIV dynamics among transgender women: a respondent driven sampling study in Rio de Janeiro, Brazil. Lancet HIV. 2017 Apr;4(4):e169–76.

13. Leite BO, Magno L, Soares F, MacCarthy S, Brignol S, Bastos FI, et al. HIV prevalence among transgender women in northeast Brazil – findings from two respondent driven sampling studies. BMC Public Health. 2022 Nov 18;22(1):2120.

14. da Rocha ABM, Barros C, Generoso IP, Bastos FI, Veras MA. HIV continuum of care among trans women and travestis living in São Paulo, Brazil. Rev Saude Publica. 54:118.

15. Jalil EM, Wilson EC, Luz PM, Velasque L, Moreira RI, Castro CV, et al. HIV testing and the care continuum among transgender women: population estimates from Rio de Janeiro, Brazil. J Int AIDS Soc. 2017 Sept 19;20(1):21873.

16. Teixeira SL, Jalil CM, Jalil EM, Nazer SC, Silva S da CC, Veloso VG, et al. Evidence of an untamed HIV epidemic among MSM and TGW in Rio de Janeiro, Brazil: a 2018 to 2020 cross-sectional study using recent infection testing. J Int AIDS Soc. 2021 June;24(6):e25743.

17. Patel P, Borkowf CB, Brooks JT, Lasry A, Lansky A, Mermin J. Estimating per-act HIV transmission risk: a systematic review. AIDS. 2014 June 19;28(10):1509–19.

18. Response to data request from the Department of HIV/AIDS, Tuberculosis, Viral Hepatitis and Sexually Transmitted Infections (Departamento de HIV/Aids, Tuberculose, Hepatites Virais e Infecções Sexualmente Transmissíveis) of the Brazilian Ministry of Health [Internet]. [cited 2025 Aug 19]. Available from: www.gov.br

19. Kohler S, Dalal S, Hettema A, Matse S, Bärnighausen T, Paul N. Human resource needs and costs for HIV pre-exposure prophylaxis provision in nurse-led primary care in Eswatini and opportunities for task sharing. Hum Resour Health. 2022 Oct 23;20(1):75.

20. Instituto Nacional de Infectologia Evando Chagas. 2024.

21. Brazilian real (BRL) to US dollar (USD) exchange rate history for 2024 [Internet]. [cited 2025 Aug 19]. Available from: https://www.exchange-rates.org/exchange-rate-history/brl-usd-2024

22. Luz PM, Deshpande V, Kazemian P, Scott JA, Shebl FM, Spaeth H, et al. Impact of pre-exposure prophylaxis uptake among gay, bisexual, and other men who have sex with men in urban centers in Brazil: a modeling study. BMC Public Health. 2023 June 13;23(1):1128.

23. Costa J de O, Ceccato M das GB, Silveira MR, Bonolo P de F, Reis EA, Acurcio F de A. Effectiveness of antiretroviral therapy in the single-tablet regimen era. Rev Saude Publica. 2018 Nov 14;52:87.

24. Meireles MV, Pascom ARP, Duarte EC, McFarland W. Comparative effectiveness of first-line antiretroviral therapy: results from a large real-world cohort after the implementation of dolutegravir. AIDS. 2019 Aug 1;33(10):1663.

25. Stellbrink HJ, Arribas JR, Stephens JL, Albrecht H, Sax PE, Maggiolo F, et al. Co-formulated bictegravir, emtricitabine, and tenofovir alafenamide versus dolutegravir with emtricitabine and tenofovir alafenamide for initial treatment of HIV-1 infection: week 96 results from a randomised, double-blind, multicentre, phase 3, non-inferiority trial. Lancet HIV. 2019 June;6(6):e364–72.

26. Sax PE, Pozniak A, Montes ML, Koenig E, DeJesus E, Stellbrink HJ, et al. Coformulated bictegravir, emtricitabine, and tenofovir alafenamide versus dolutegravir with emtricitabine and tenofovir alafenamide, for initial treatment of HIV-1 infection (GS-US-380–1490): a randomised, double-blind, multicentre, phase 3, non-inferiority trial. The Lancet. 2017 Nov 4;390(10107):2073–82.

27. Spizzirri G, Eufrásio R, Lima MCP, de Carvalho Nunes HR, Kreukels BPC, Steensma TD, et al. Proportion of people identified as transgender and non-binary gender in Brazil. Sci Rep. 2021 Jan 26;11(1):2240.

**Supporting information Table A1.** Additional model inputs for a cost-effectiveness analysis of LA PrEP cost-effectiveness of MSM and TGW.

|  | **Value** | **Reference** |
| --- | --- | --- |
| **HIV care** | | |
| Increase in CD4 count after 48 weeks on suppressive ART, cells/µL, mean (SD) |  | [22] |
| First-line (TLD) |  |  |
| First two months | 80.4 (30) |  |
| After Month 2 | 4.02 (1.5) |  |
| Second-line (DRV/r + 2NRTIs) |  |  |
| First 2 months | 83.2 (38.175) |  |
| After month 2 | 4.02 (1.5) |  |

| Suppression at 12 months, % |  | Der. from [23,24] |
| --- | --- | --- |
| First-line (TLD) | 88 |  |
| Second-line (DRV/r+2NRTIs) | 73 |  |
| Rate of virologic failure, incidence/100 person-months |  | Der. from [25,26] |
| First-line (TLD) | 0.35 |  |
| Second-line (DRV/r+2NRTIs) | 0.93 |  |
| Rate of disengagement from HIV care/ 100 person-years |  | INI^†^ |
| MSM | 11.6 |  |
| TGW | 27.3 |  |

**Supporting information Table A1, continued.** Additional model inputs for a cost-effectiveness analysis of LA PrEP cost-effectiveness of MSM and TGW.

|  | | | **Value** | **Reference** |
| --- | --- | --- | --- | --- |
| **MSM HIV care continuum in 2021** | | | |  |
| MSM with HIV, N | 476,530 | | Derived from [4–9] |  |
| On ART among MSM with HIV, % | 64% | |  |  |
| MSM without HIV, n | 1,357,050 | |  |  |
| New infections, n | 29,750 | | Derived from [4–10] |  |
| **TGW HIV care continuum in 2021** | | | |  |
| TGW with HIV, n | 384,950 | | Derived from [4,5,12–15,27] |  |
| Percentage on ART among TGW with HIV, % | 59% | |  |  |
| TGW without HIV, n | 679,960 | |  |  |
| New infections, n | 21,480 | | Derived from [4,5,10,12–16,27] |  |
| **Cost** | | | |  |
| HIV viral load test, $/test | 12.27 | | [18,21] |  |
| CD4 count test, $/test | 10.84 | | [18,21] |  |

**Supporting information Table A1, continued.** Additional model inputs for a cost-effectiveness analysis of LA PrEP cost-effectiveness of MSM and TGW.

|  | **Value** | **Reference** |
| --- | --- | --- |
| Acute | 100.72 | [1–10] |
| >100,000 copies/mL | 14.54 |  |
| 10,000-100,000 copies/mL | 13.07 |  |
| 3,000-10,000 copies/mL | 6.71 |  |
| 20-3,000 copies/mL | 3.32 |  |
| ≤20 copies/ML | 0 |  |
| Acute | 8.03 | [1–10,17] |
| >100,000 copies/mL | 1.16 |  |
| 10,000-100,000 copies/mL | 1.04 |  |
| 3,000-10,000 copies/mL | 0.53 |  |
| 20-3,000 copies/mL | 0.26 |  |
| ≤20 copies/ML | 0 |  |

| Abbreviations: ART, antiretroviral therapy; DRV/r, darunavir/ritonavir; LA PrEP, long-acting pre-exposure prophylaxis; MSM, men who have sex with men; NRTIs, nucleoside reverse transcriptase inhibitors; SD, standard deviation, TGW, transgender women; TLD, tenofovir disoproxil, lamivudine, and dolutegravir |
| --- |

**Supporting information Table A2.** Results of an analysis of LA PrEP cost-effectiveness for MSM and TGW in Brazil under a pessimistic scenario: partial substitution coverage (15% oral PrEP and 15% LA PrEP for a total coverage of 30%)

| Strategy | Undisc.  LE, y | Disc.  LE, y | 10-year  infection risk, % | Lifetime  infection risk^‡^, % | Undisc. costs, $ | Disc.  costs, $ | ICER^†^, $/YLS |
| --- | --- | --- | --- | --- | --- | --- | --- |
| **MSM** | | | | | | | |
| LA PrEP option = cabotegravir |  |  |  |  |  |  |  |
| *SOC* | 39.0 | 16.2 | 14.0 | 21.4 | 2,800 | 1,060 | - |
| *SOC+CAB-LA* | 39.2 | 16.3 | 12.3 | 18.9 | 4,210 | 1,990 | 9,320 |
| LA PrEP option = lenacapavir |  |  |  |  |  |  |  |
| *SOC* | 39.0 | 16.2 | 14.0 | 21.4 | 2,800 | 1,060 | - |
| *SOC+LEN-LA* | 39.2 | 16.3 | 12.2 | 18.7 | 4,130 | 1,950 | 8,570 |
| **TGW** | | | | | | | |
| LA PrEP option = cabotegravir |  |  |  |  |  |  |  |
| *SOC* | 36.0 | 15.7 | 20.0 | 29.5 | 2,660 | 1,080 | - |
| *SOC+CAB-LA* | 36.5 | 15.8 | 17.7 | 26.1 | 4,050 | 1,990 | 8,620 |
| LA PrEP option = lenacapavir |  |  |  |  |  |  |  |
| *SOC* | 36.0 | 15.7 | 20.0 | 29.5 | 2,660 | 1,080 | - |
| *SOC+LEN-LA* | 36.5 | 15.8 | 17.5 | 25.8 | 3,980 | 1,950 | 7,840 |
| †IThis is the discounted ICER of SOC+LA compared to SOC, where the cost of CAB-LA or LEN-LA is $600/year, adjusted to include the benefit of reduced primary transmissions. Without adjusting for transmissions, the discounted ICERs would be $16,210/YLS (MSM) and $9,240/YLS (TGW) for SOC+CAB-LA versus SOC, or $15,470/YLS (MSM) and $8,430/YLS (TGW) for SOC+LEN-LA versus SOC.  ‡We did not model HIV incidence above age 50.  Abbreviations: Disc., discounted; ICER, incremental cost-effectiveness ratio; LE, life expectancy; LA PrEP, long-acting pre-exposure prophylaxis; MSM, men who have sex with men; SOC, standard-of-care using oral PrEP at 20% coverage; SOC+LA, strategy providing both oral PrEP (coverage 15%) and LA PrEP (coverage 15%) using CAB-LA or LEN-LA; TGW, transgender women; Undisc., undiscounted | | | | | | | |

**FIGURE LEGENDS**

**Supporting information Figure A1. Maximum costs of LA PrEP at varying willingness-to-pay thresholds.**

The plot displays the maximum annual cost of LA PrEP at which a strategy that uses LA PrEP at 20% coverage in combination with oral PrEP at 20% coverage (*SOC+LA*) would remain cost-effective compared to a strategy that uses only oral PrEP at 20% coverage (*SOC*) for MSM in Brazil for a range of willingness-to-pay thresholds. The horizontal dashed lines at $5,020/YLS and $8,740/YLS represent 0.5x and 0.87x Brazil GDP per capita, respectively. The vertical dashed lines cross the x-axis at the maximum costs for which each strategy would be cost-effective at these willingness-to-pay thresholds.

Abbreviations: CAB-LA, cabotegravir long-acting; GDP, gross domestic product; ICER, incremental cost-effectiveness ratio; LA PrEP, long-acting pre-exposure prophylaxis; LEN-LA, lenacapavir long-acting; MSM, men who have sex with men; YLS, years of life saved.

**Supporting information Figure A2. One-way sensitivity analyses of LA PrEP cost-effectiveness for TGW in Brazil.**

The tornado diagram displays the impact of varying individual parameters on the change in ICERs (*SOC+CAB-LA* (dark blue), *SOC+LEN-LA* (light blue) versus SOC*).* Each horizontal bar displays the range of ICERs that result from varying a single parameter. For each parameter, the base case value is listed first, followed by the range in the parentheses. The base case values would lead to the thin vertical lines through the diagram, where the ICERs are $6,790/YLS (*SOC+CAB-LA)* and $6,360/YLS for (*SOC+LEN-LA)*. The left value in the parentheses would lead to the lower ICER and the right value would lead to the higher ICER. A longer bar reflects a greater change in ICER as the parameter was varied. The black dotted vertical line at $8,740/YLS marks the willingness-to-pay threshold. The bars that extend to the right of this line mark where *SOC+LA* would not be cost-effective compared to *SOC*, while the bars that extend to the left mark where *SOC+LA* would be cost-effective compared to *SOC*.

Abbreviations: CAB-LA, cabotegravir long-acting; ICER, incremental cost-effectiveness ratio; LA PrEP, long-acting pre-exposure prophylaxis**;** LEN-LA, lenacapavir long-acting; TGW, transgender women; YLS, years of life saved.

**Supporting information Figure A3. Multiway sensitivity analyses of LA PrEP cost-effectiveness for MSM in Brazil at varying LA PrEP costs and levels of HIV incidence among MSM aged 18-29 years and 18-49 years.**

This figure presents a multiway sensitivity analysis on the ICER ($/YLS) of *SOC+LA* versus *SOC* and annual CAB-LA (**panel A**) and LEN-LA (**panel B**) cost ($/year) at three levels of HIV incidence among MSM 18-29 years (solid) and 18-49 years (dashed): 0.5x, 1x, and 2x base case values (3.37%/year [18-29 years]/1.11%/year [30-49 years]). The vertical axis reports the ICER of *SOC+LA* vs. *SOC*. The horizontal axis reports the price of LA PrEP in *SOC+LA*. The dashed horizontal line at $8,740/YLS in both panels represents the willingness-to-pay threshold. When the ICER falls below this threshold, *SOC+LA* would be cost-effective compared to *SOC*.

Abbreviations: CAB-LA, cabotegravir long-acting; ICER, incremental cost-effectiveness ratio; LA PrEP, long-acting pre-exposure prophylaxis**;** LEN-LA, lenacapavir long-acting; MSM, men who have sex with men; YLS, years of life saved.

**Supporting information Figure A4. Multiway sensitivity analyses of LA PrEP cost-effectiveness for MSM in Brazil at varying levels of HIV incidence and LA PrEP costs, for three distributions of PrEP coverage in the *SOC+LA* strategy with 30% total PrEP coverage**

This figure presents a multiway sensitivity analysis on the ICER ($/YLS) of *SOC+CAB-LA* versus *SOC* (**panel A**) and *SOC+LEN-LA* **(panel B**) versus *SOC* when varying three parameters simultaneously: LA PrEP cost, HIV incidence among MSM 18-49 years, and oral vs. LA coverage in *SOC+LA*. The overall PrEP coverage in *SOC+LA* was held constant at 30%. From left to right, each of the three matrices represents a different ratio of oral to LA PrEP coverage in *SOC+LA*: [1:4], [1:1], and [4:1]. Within each matrix, the horizontal shows three levels of HIV incidence among MSM 18-49 years: 0.5x, 1x, and 2x base case values (3.37%/year [18-29 years]/1.11%/year [30-49 years]). The vertical shows four annual costs of LA-PrEP ($/year): 300, 600, 900, and 1,200. Each cell in the matrices shows the ICER for a scenario with the specified PrEP distribution in *SOC+LA*, HIV incidence for MSM 18-49 years, and LA PrEP cost. Different shades of blue represent scenarios where *SOC+LA* would be cost-effective compared to *SOC*, with darker blue corresponding to ICERs below 0.5x GDP, or $5,020/YLS, and light blue corresponding to ICERs between 0.5x GDP and the willingness-to-pay threshold of 0.87x GDP, or $8,740/YLS. The light and darker red cells represent scenarios where *SOC+LA* would not be cost-effective compared to SOC, with light red corresponding to ICERs between 0.87x GDP and 1.2x GDP, or $8,740-$12,060/YLS, and darker red corresponding to ICERs above 1.2x GDP, or $12,060/YLS.

Abbreviations: CAB-LA, cabotegravir long-acting; GDP, gross domestic product; ICER, incremental cost-effectiveness ratio; LA PrEP, long-acting pre-exposure prophylaxis; LEN-LA, lenacapavir long-acting; MSM, men who have sex with men; y, year; YLS, years of life saved.

**Supporting information Figure A5. Multiway sensitivity analyses LA PrEP cost-effectiveness for TGW in Brazil at varying levels of HIV incidence and LA PrEP costs, for six distributions of PrEP coverage in the *SOC+LA* strategy**

This figure presents a multiway sensitivity analysis on the ICER ($/YLS) of *SOC+CAB-LA* versus *SOC* (**panel A**) and *SOC+LEN-LA* **(panel B**) versus *SOC* when varying four parameters simultaneously: LA PrEP cost, HIV incidence among TGW 18-49 years, overall PrEP coverage, and oral vs. LA coverage in *SOC+LA*. From left to right, each of the three matrices represents a different ratio of oral to LA PrEP coverage in *SOC+LA*: [1:4], [1:1], and [4:1]. Within each matrix, the horizontal shows three levels of HIV incidence among TGW 18-49 years: 0.5x, 1x, and 2x base case values (5.1%/year [18-29 years]/1.7%/year [30-49 years]). The vertical shows four annual costs of LA PrEP ($/year): 300, 600, 900, and 1,200. Each cell in the matrices shows the ICER for a scenario with the specified PrEP distribution in *SOC+LA*, HIV incidence for TGW 18-49 years, and LA PrEP cost. Different shades of blue represent scenarios where *SOC+LA* would be cost-effective compared to *SOC*, with dark blue corresponding to ICERs below 0.5x GDP, or $5,020/YLS, and light blue corresponding to ICERs between 0.5x GDP and the willingness-to-pay threshold of 0.87x GDP, or $8,740/YLS. The light and darker red cells represent scenarios where *SOC+LA* would not be cost-effective compared to SOC, with light red corresponding to ICERs between 0.87x GDP and 1.2x GDP, or $8,740-$12,060/YLS, and darker red corresponding to ICERs above 1.2x GDP, or $12,060/YLS.

Abbreviations: CAB-LA, cabotegravir long-acting; GDP, gross domestic product; ICER, incremental cost-effectiveness ratio; LA PrEP, long-acting pre-exposure prophylaxis; LEN-LA, lenacapavir long-acting; TGW, transgender women; YLS, years of life saved.

**
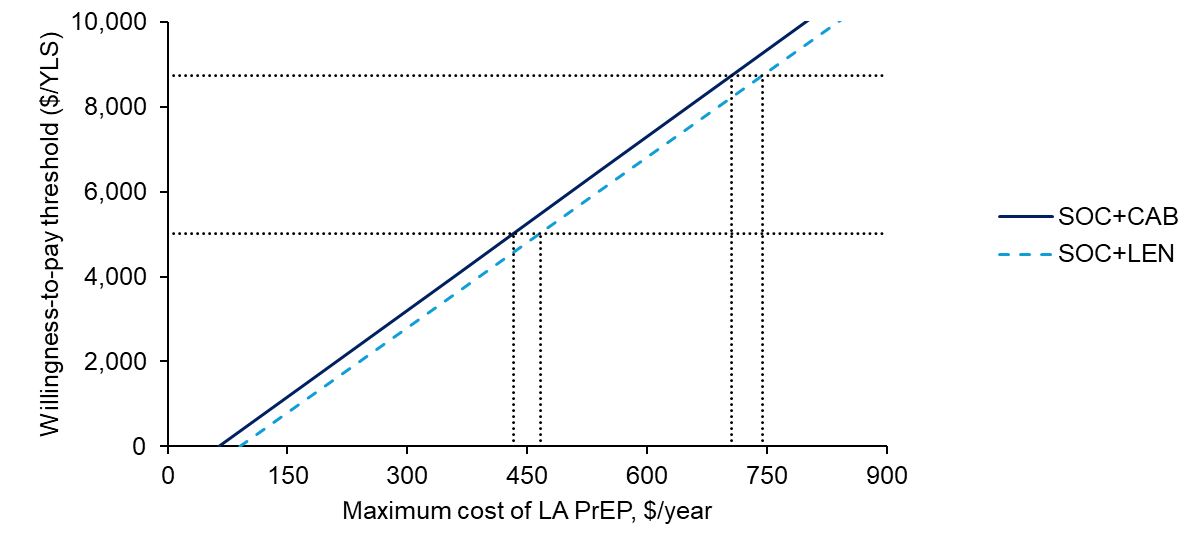
**

**Supporting information Figure A1.**

**
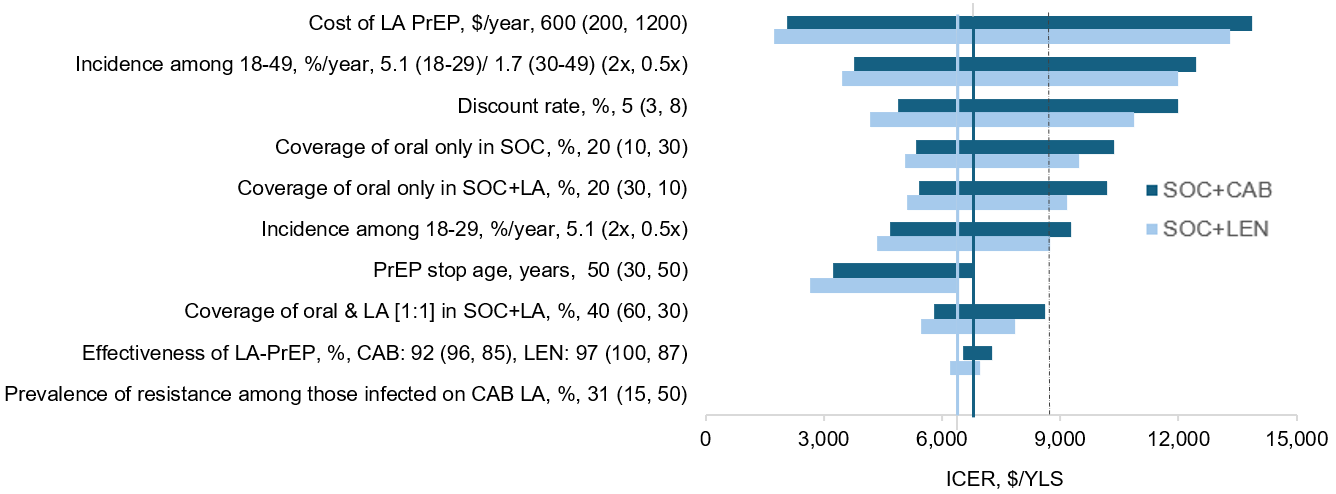
**

**Supporting information Figure A2.**

**B**

**Supporting information Figure A3**

**Supporting information Figure A4.**

**Supporting information Figure A5.**
